# Differences in gut bacterial community composition between modern and slower-growing broiler breeder lines: Implications of growth selection on microbiome composition

**DOI:** 10.3389/fphys.2023.1151151

**Published:** 2023-03-21

**Authors:** Naama Shterzer, Yara Sbehat, Binita Poudel, Nir Rothschild, Olanrewaju Eunice Oloko, Joseph Headrick, Erik Petersen, Shelly Druyan, Erez Mills

**Affiliations:** ^1^ Department of Animal Sciences, Robert H. Smith Faculty of Agriculture, Food, and Environment, The Hebrew University of Jerusalem, Rehovot, Israel; ^2^ Agricultural Research Organization, Volcani Center, Department of Poultry and Aquaculture Science, Rishon LeTsiyon, Israel; ^3^ Department of Health Sciences, College of Public Health, East Tennessee State University, Johnson City, TN, United States

**Keywords:** akkermansia, gut microbiome, broiler breeders, breeding program, genetics microbiome interaction

## Abstract

In the last century broiler chicken lines have undergone an extensive breeding regime aimed primarily at growth and high meat yield. It is not known if breeding has also resulted in a change to the broiler breeder’s associated gut microbiota. Here we compared the gut microbiota of 37-week-old commercial Cobb breeding dams with dams from a broiler Legacy line which has not undergone selection since 1986. The dams from both lines were kept together in the same shed under the same management protocol from day of hatch to avoid additional confounders. We chose this age to allow significant bacterial exchange, thus avoiding exposure dependent artifacts and so that we could compare dams at the same developmental state of adulthood and peak laying performance. Significant differences in the composition of the cecum bacterial communities were found. Bacteria of the genus *Akkermansia*, implicated in mucin degradation and associated with host metabolic health, accounted for 4.98% ± 5.04% of the Cobb cecum community, but were mostly absent from the ceca of the Legacy line dams. Inversely, Legacy dams had higher levels of Clostridiales, Lactobacillales and Aeromonadales. These results show that breeding has resulted in a change in the gut microbiota composition, likely by changing the physiological conditions in the mucosa. It remains unclear if changes in gut microbiota composition are a part of the mechanism affecting growth or are a secondary result of other physiological changes accelerating growth. Therefore, the identification of these changes opens the door to further targeted research.

## Introduction

Breeding programs primarily targeting growth and high meat yield have successfully transformed broiler lines in the last decades by substantially increasing growth (Zuidhof et al., 2014). While many physiological effects of breeding programs are known, such as changes to metabolism and the intestinal tract, including an increase of surface area (Mitchell and Smith, 1991; Zuidhof et al., 2014; Tallentire et al., 2016), it remains unclear if the gut microbiota has been modulated by breeding programs. The aim of this study is to address this point by comparing the cecum microbiota of a current modern commercial breeding dam line and a legacy broiler line, which has not undergone any selection since 1986 (Yair et al., 2017; Ben-Gigi et al., 2021).

It can be hypothesized that the gut microbiota is likely to be affected by physiological changes introduced in the host through breeding programs. These changes can include differences in retention time, affecting microbial clearance (Rougière and Carré, 2010). Differences in mucin expression levels can also affect the gut microbiota since bacteria use mucin as a binding site or as a nutrient (Cheled-Shoval et al., 2014). Other factors include reduced nutritional availability due to changes in host absorption (Croom et al., 1999; Schmidt et al., 2009), and changes in the regulation of components of the immune system, such as changes in secretion of antimicrobial peptides or IgA into the gut lumen (Qureshi and Havenstein, 1994; Schokker et al., 2015).

The gut microbiota can affect host growth. For example, different gut microbial communities can induce host obesity (John and Mullin, 2016) but can also reduce weight (von Schwartzenberg et al., 2021). One mechanism by which gut bacteria can positively affect growth is by converting indigestible fibers into short-chain fatty acids which the host can absorb and utilize (Krajmalnik-Brown et al., 2012). However, gut bacteria are also potential competitors and can reduce nutrient availability for the host (Romano et al., 2017). Finally, the gut microbiota can also affect the maturation and development of the host’s intestinal tract, thereby affecting its ability to utilize the feed (Hutsko et al., 2016; Dougherty et al., 2020). Thus, theoretically, there are multiple mechanisms by which modulation of gut microbiota composition during breeding programs may have supported or opposed the target of fast growth and higher meat yield.

Previous studies have examined the relationship between broiler lines and gut microbiota composition. A comparison of the cecal (Richards et al., 2019) and ileal (Richards-Rios et al., 2020) microbiomes of three fast growing commercial broiler lines up to day 42 revealed differences only on the day of hatch and day 3 of life. Thus, while some differences were observed early on, possibly because of different chick sources, the composition over time converged, likely because the chicks were raised together and exchanged gut microbes. This implies that fast growing commercial broiler lines are similar in their interaction with their gut microbiota at least until the age of 42 days. In comparison, studies comparing fast growing broiler lines to a historic line, or a line selected for slow growth, were able to identify differences in ileal and fecal bacterial communities. Lumpkins et al. (2010) identified composition differences between the ileal bacterial community of a historic line and two commercial modern broiler lines in the first 35 days of life, and Zhao et al. (2013) identified multiple composition differences in fecal samples at the age of 245 days of two divergent lines, selected for 54 generations for high or low body weight. Finally, a recent study comparing the ileal microbiota of four different lines of fast and slow growing broilers including the ancestral Jungle Fowl at the age of 56 days found unique signatures for the different lines and predicted microbiota functions (Emami et al., 2022). Here we extend these studies by comparing the cecum bacterial community of breeder dams from a commercial broiler line and a Legacy line. As the cecum is the site of bacterial fermentation in chickens, any relevant effect of the gut community on poultry growth would likely occur at the cecum. Furthermore, differences in adult breeders might be easier to detect because the microbiota has stabilized, they might reveal physiological differences that are relevant to younger birds, and they might affect the fertility as well as egg laying efficiency of breeders, including the nutrients deposited into the egg.

Thus, to examine the interaction of genetics and microbiota composition in the context of breeding programs, we raised modern Cobb breeder dams alongside dams from a Legacy line, which was kept as a relaxed line (without selection) from 1986 (Yair et al., 2017; Ben-Gigi et al., 2021) and compared their cecum bacterial communities.

## Materials and methods

### Genetic lines

All animal trials were conducted in accordance with the guidelines of the National Council for Animal Experimentation and were subjected to approval by the Hebrew University of Jerusalem’s Ethics committee, approval No. AG-19-15897-3.

Two genetic lines were utilized: Cobb—the current Cobb breeder line, and Legacy—a local Israeli broiler line which has not been under selection pressure since 1986 (Yair et al., 2017; Ben-Gigi et al., 2021).

### Growth conditions and confounder avoidance

Eggs from both lines were incubated and hatched on site. Sixty-two Cobb breeders and 84 Legacy breeders were kept in the same shed, under the same conditions and handled by the same individuals from hatch and throughout the experiment. Birds were placed in individual cages (45 × 45 cm) at 6 weeks of age. All the birds were raised according to the same breeder management protocol (Cobb-Vantress, 2018), including the same feed. During the production stage, birds were fed once a day in the morning according to the feeding tables in the management protocol. At the age of 24 weeks the birds were transferred to cages in an open shed and were exposed to 16 h of light per day. Eggs were collected manually twice a day, and individual laying was monitored. Sampling was done at the age of 37 weeks, after both lines have reached their peak laying state and were still producing at high levels (Supplementary Figure S1). At the age of 37 weeks, it is assumed that there was ample time for microbial exchange between animals to offset any differences in initial exposure. Furthermore, by waiting until adulthood and peak laying status, we were ensuring that differences between the birds are not an artifact of different effective physiological age.

### Sample collection

At age 37 weeks, ten animals of each line were randomly selected, weighed and then euthanized by cervical dislocation. Cecum samples were removed, their contents were emptied out into 5 mL of sterile PBS and flash frozen with liquid nitrogen. All samples were stored at −20°C until processing. One sample of a Legacy dam was contaminated during the sampling procedure, and therefore was removed from the microbiota analyses.

### Sampling of other broiler and broiler breeder sources

To determine the presence of *Akkermansia* in other broilers, four more sources of modern broilers and broiler breeders were sampled. Broiler source #1—ceca of six Ross breed broilers were sampled from a commercial farm at age 32 days; Broiler source #2—ceca of five Ross breed broilers were sampled from a commercial farm at age 34 days; Broiler breeders source #1—ceca of five Ross breed broiler breeders were sampled from the experimental farm in the Hebrew University of Jerusalem’s Faculty of Agriculture at age 55 weeks (Shterzer et al., 2022); Broiler breeders source #2—ceca of five Ross breed broiler breeders were sampled from a commercial farm at age 56 weeks. All ceca were sampled as mentioned above.

### DNA extraction

DNA was extracted by mixing 700 µL of sample with 700 µL of Tris-saturated phenol and 100 µL of 10% SDS. The mixture was disrupted with 0.1 mm glass beads followed by phenol-chloroform extraction, as described previously (Stevenson and Weimer, 2007). Briefly, the aqueous phase was extracted twice with phenol, then twice with a phenol-chloroform mixture (1:1) and finally twice with chloroform. DNA was subsequently precipitated with isopropanol and suspended in double distilled water.

### 16S rRNA gene sequencing

16S rRNA gene library was prepared and sequenced according to the Earth Microbiome Project protocol (Thompson et al., 2017) using V4 primers 515F (GTGYCAGCMGCCGCGGTAA) and 806R (GGACTACNVGGGTWTCTAAT). 250 bp paired-end sequencing was carried out on an Illumina Miseq platform using a V2 reagent kit by Hylabs (Rehovot, Israel). Sequence processing and taxonomy assignment were performed using Quantitative Insights Into Microbial Ecology (QIIME2) version 2020.11.1 (Bolyen et al., 2019) as described previously (Shterzer et al., 2020). Briefly, amplicon sequence variants (ASVs) were determined with Dada 2 plugin version 2020.11.1 (Callahan et al., 2016) using the denoise-paired method, which filters out reads with estimated number of errors >2. All reads were truncated at position 200; otherwise, default parameters were used. After denoising, a total of 313,070 reads were retained, with 16,477 ± 3,945 reads per sample (min—9,691; max—28,241). ASVs with under five reads were discarded and all samples were normalized to 4,000 reads per sample with the feature-table plugin using the rarefy method (Weiss et al., 2017). Taxonomy was assigned using a naive-bayes classifier (Pedregosa et al., 2011) trained on the Greengenes database (McDonald et al., 2012). All ASVs with the taxonomic assignment of “Bacteria” were compared to the NT database using BLAST (Altschul et al., 1990) and removed if they were 100% identical to *Gallus* mitochondrion.

### Statistical analysis

ANCOM analysis was implemented using QIIME2 (Mandal et al., 2015) to identify differential abundance of phylogenetic groups in all levels (phylum, class, order, family, genus and species). To that end, the ASV feature table was collapsed at different taxonomic levels and ANCOM analysis was performed on the collapsed tables, as well as on the original ASV-level table. To test the significance of the differences in microbiome composition between the lines, ANOSIM test was performed using Past 4.05 (Hammer et al., 2001). Otherwise, all statistical tests (Welch’s *t*-test, Mann-Whitney and Spearman’s rank correlation) were performed with GraphPad Prism 8.0.0 (GraphPad Software, San Diego California United States of America, www.graphpad.com).

## Results

### Weight comparison between the Cobb and Legacy breeder dams

**FIGURE 1:**
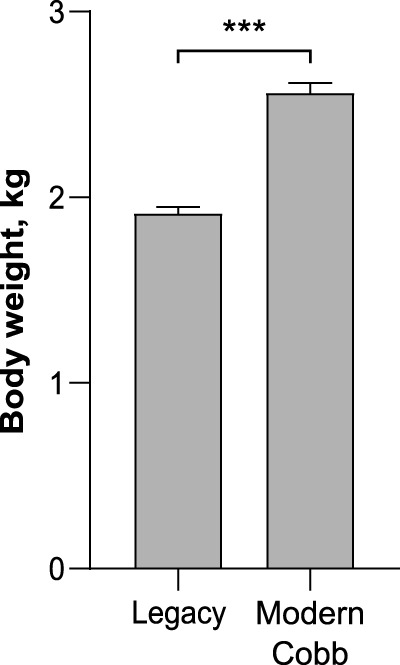
Body weight of Legacy line and Cobb line individuals. Data are presented as mean ± SEM; *n* = 10; Welch’s *t*-test; ***p < 0.0001.

While marketing age poultry of modern and Legacy lines are very different in size, breeders are kept on a strict diet as to avoid obesity which will negatively affect their laying ability (Zuidhof et al., 2017). To quantify the weight differences between breeders of both lines we weighed the birds before sampling their microbiota. Indeed, on week 37 Cobb dams were 34% heavier than Legacy dams (Welch’s *t*-test *p* < 0.0001; Figure 1).

### Diversity analysis of the microbiome of Cobb and Legacy dams

**FIGURE 2:**
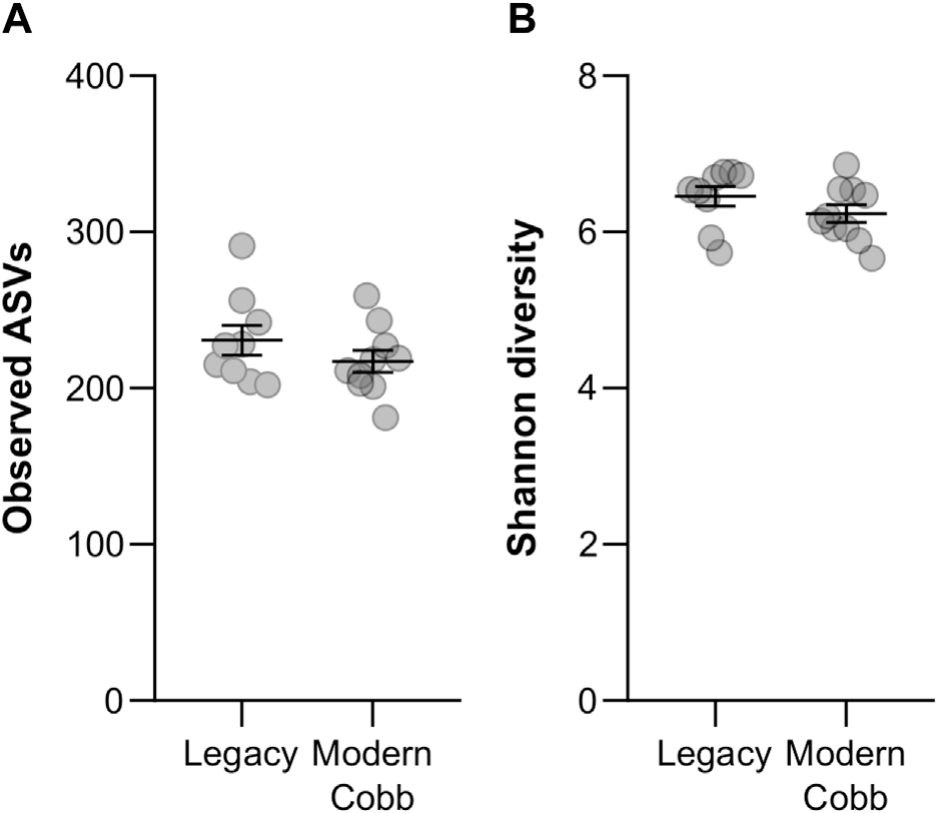
Alpha-diversity measures. Richness **(A)** and Shannon diversity **(B)** of Legacy line and Cobb line individuals. Data are presented as mean ± SEM; Legacy *n* = 9, Cobb *n* = 10; Mann-Whitney test; *p* > 0.05.

To compare the gut community profile of the two lines, we collected samples of cecum contents and performed 16S rRNA gene sequencing to characterize the bacterial community. A comparison of the number of observed ASVs between Cobb and Legacy dams showed similar richness in the cecum communities (Mann-Whitney test *p* > 0.05; Figure 2A). An analysis of Shannon diversity, also integrating evenness measures, revealed the same trend (Mann-Whitney test *p* > 0.05; Figure 2B). Thus, regarding alpha-diversity measures of richness and evenness the cecum communities of the two lines are similar.

### Dissimilarity analysis of the microbiome of Cobb and Legacy dams

**FIGURE 3:**
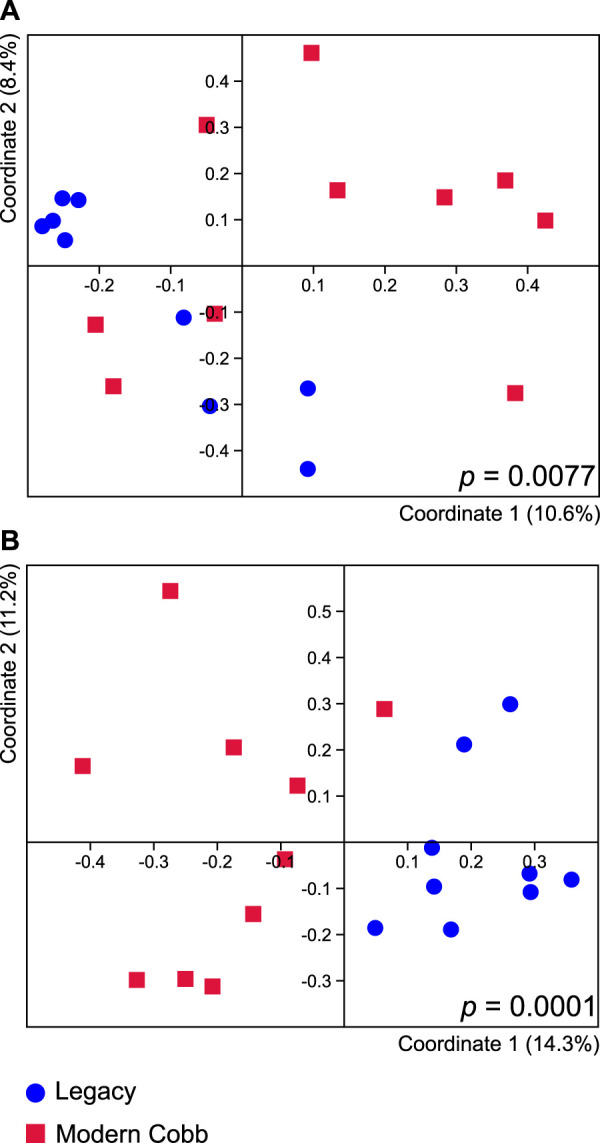
Cecal microbiome differences between Legacy line and Cobb line individuals. PCoA analysis of Legacy line and Cobb line using Jaccard **(A)** and Bray-Curtis **(B)** metrics. ANOSIM *p* values for each metric are indicated in the figure. Legacy *n* = 9, Cobb *n* = 10.

Dissimilarity analysis utilizing Jaccard index showed a significant difference in the cecum communities of Cobb and Legacy dams (ANOSIM *p* = 0.0077; Figure 3A). A similar analysis utilizing Bray-Curtis index showed a difference with a greater statistical significance (ANOSIM *p* = 0.0001; Figure 3B). As Jaccard index is based solely on presence and absence of specific ASVs, whereas Bray-Curtis also integrates relative abundance data, this implies that differences between the cecum communities of Cobb and Legacy dams are based on both the ability of specific strains to colonize the different lines as well as their ability to grow to large numbers and perhaps compete with other parts of the microbial community.

### Composition analysis of the cecum microbiome of Cobb and Legacy dams

**FIGURE 4:**
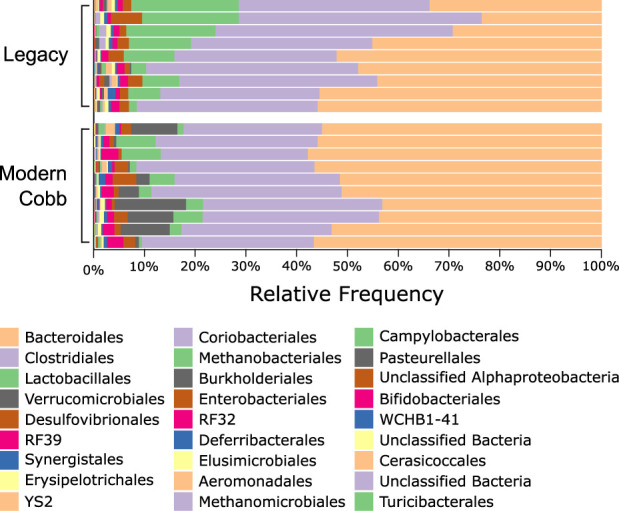
Taxonomic composition of Legacy line and Cobb line Cecal microbiome at the Order level.

**FIGURE 5:**
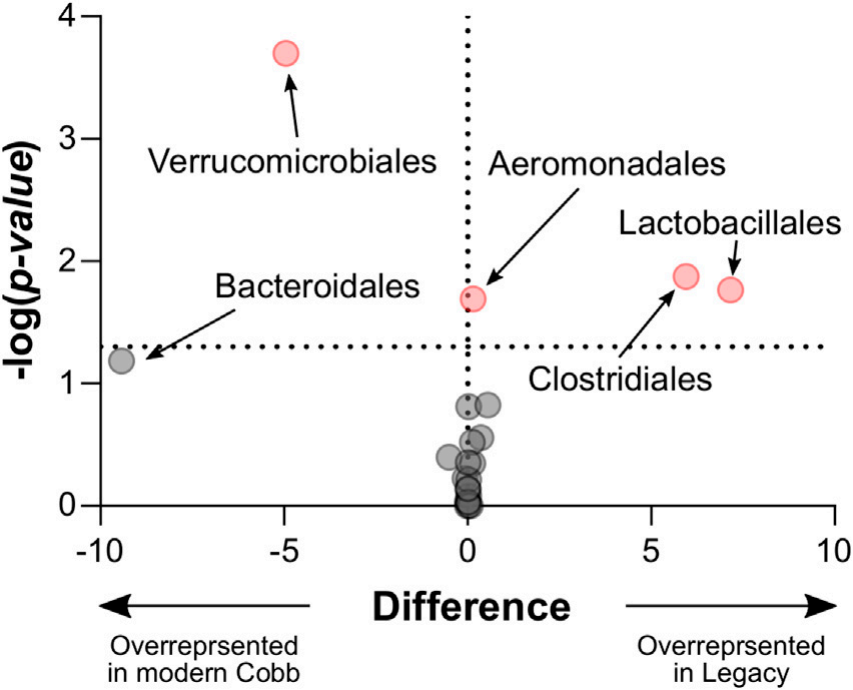
Volcano plot of Mann-Whitney results for all taxonomic orders. Significantly different orders are marked red. Legacy n = 9, Cobb n = 10.

**FIGURE 6:**
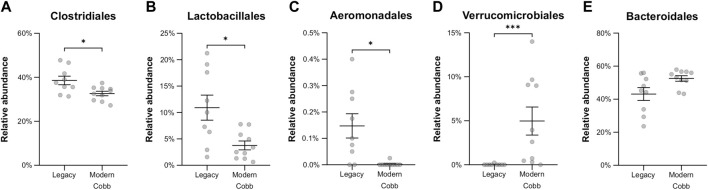
Relative abundance of orders that were significantly different between the Legacy and Cobb lines. **(A)** Clostridiales **(B)** Lactobacillales **(C)** Aeromonadales **(D)** Verrucomicrobiales **(E)** Bacteroidales. Data are presented as mean ± SEM. Legacy n = 9, Cobb n = 10; Mann-Whitney test; *p ≤ 0.05, ***p ≤ 0.001.

An analysis of the cecum communities at the order level revealed, as expected, that the two most abundant orders in the cecum samples were Bacteroidales and Clostridiales for both dam lines (Figure 4). An analysis of differential abundance using ANCOM (Mandal et al., 2015) identified differences between Cobb and Legacy dams in all phylogenetic levels from phylum down, all of them belonging to the lineage of the genus *Akkermansia* (phylum Verrucomicrobia, class Verrucomicrobiae, order Verrucomicrobiales, family Verrucomicrobiaceae, and genus *Akkermansia*; Supplementary Data Sheet S1). *Akkermansia* was the only member in the Verrucomicrobiales order present in our dataset and accounted for 4.98% ± 5.04% of the microbiota in Cobb dams, while in Legacy dams it was absent in all but one individual (in which it had a relative abundance of 0.22%). This order was also significantly different by Mann-Whitney test (*p* = 0.0002). Other significantly different bacterial orders identified by Mann-Whitney test are Clostridiales (*p* = 0.0133), Lactobacillales (*p* = 0.0172) and Aeromonadales (*p* = 0.0204), which are higher in Legacy dams (Figure 5; Figure 6). *Akkermansia* levels were significantly negatively correlated to Lactobacillales and Aeromonadales levels, and Lactobacillales levels were also found to be negatively correlated to Bacteroidales levels (Table 1).

**TABLE 1:** Spearman Correlation between phylogenetic groups.

Group 1	Group 2	*p-value*	r value
*Akkermansia*	Lactobacillales	0.0039	−0.6287
	Aeromonadales	0.0302	−0.4976
	Clostrediales	0.1199	−0.3691
Bacteroidales	Lactobacillales	0.0124	−0.5614

### *Akkermansia* incidence in sampled chicken communities

**FIGURE 7:**
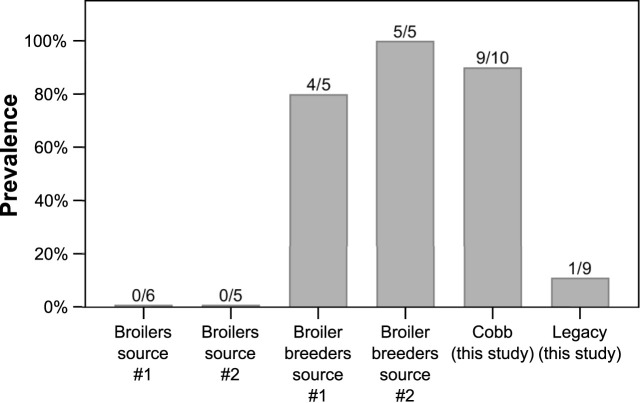
Prevalence of *Akkermansia* in previously sampled datasets. Data are presented as percentage, and the number of birds with *Akkermansia* out of total birds in each dataset is indicated above the bars.

To understand the relevance of *Akkermansia* levels in Cobb dams, we studied the incidence of the genus *Akkermansia* in the ceca of other groups of chickens from a modern broiler line sampled over the last few years (Figure 7). *Akkermansia* was found in two other groups of adult broiler breeders that we previously sampled. In two groups of younger slaughter aged broilers, *Akkermansia* was not represented, i.e., no reads of ASVs annotated as *Akkermansia* were identified. Thus, adults of modern lines were colonized by *Akkermansia*, while adults of the Legacy line and younger modern broilers were not.

## Discussion

To determine if breeding programs also modulated the gut microbiota, dams from the current Cobb commercial line, which targets high growth and meat yield, were compared with dams from a Legacy line that had not undergone targeted selection since 1986. Indeed, in the 35 years that have passed, the primarily growth targeted breeding program had a substantial effect on chicken size even under a feed restricted diet. This change in total weight is likely accompanied with multiple physiological changes that can affect gut microbiota community composition (Mitchell and Smith, 1991; Zuidhof et al., 2014; Tallentire et al., 2016).

To avoid confounding factors, we sampled Cobb and Legacy breeder dams that were housed together from day of hatch. This way, all dams were subjected to the exact same environmental conditions, including temperature, exposure to pathogens, feed (Havenstein et al., 2003), and handlers. When sampling age-matched animals that grow at different rates, any identified differences might be a result of the different developmental states. We avoided this confounder by sampling at an age when both breeds have reached peak laying performance and are well into adulthood. Last, by allowing the two lines to grow together for 37 weeks, we have allowed enough time to pass for multiple microbial cross contamination events between birds to occur. By removing these confounders, we have ensured that identified differences in microbiota composition are likely to be a result of gut environment differences due to differences in genetics.

The effects of breeding programs targeting growth on host physiology are still being assessed. Apart from the positive effects, including growth itself, a number of negative effects have been identified, including skeletal defects, metabolic disorders and altered immune function (reviewed by Zuidhof et al., 2014). Our results show a change in the cecum community composition between a current modern breeding line and the Legacy line. These results add to a previous report showing differences in the ileal bacterial community of a historic line and two commercial modern broiler lines (Lumpkins et al., 2010), and another report showing unique bacterial signatures for four different fast and slow growing broilers, including the ancestral Jungle Fowl (Emami et al., 2022). Thus, it can be concluded that genetic changes introduced during the breeding program resulted in a change in the gut bacterial community. These results raise an interesting question: are these genetic and physiologic differences between the two lines a direct result of a breeding program aimed at fast growth and meat yield, or did they happen by chance? The most prominent difference in the cecum community was that *Akkermansia* genus was a relatively high abundance member in the Cobb dams, while in the Legacy dams it was mostly absent (Figure 6). Interestingly, a high prevalence of *Akkermansia* was also found in Ross breeders (Figure 7). Assuming the breeding programs that gave rise to the current Cobb and Ross breeds are independent, these results might imply that *Akkermansia* in breeders is associated with fast growth and high meat yield phenotypes. Further research is required to establish this association.

*Akkermansia* bacteria are interesting as they are also found in humans and are studied as a future probiotic strain (Naito et al., 2018). These bacteria are mucin degraders and have been inversely correlated with metabolic disease in humans and mice (Everard et al., 2013; Naito et al., 2018). It was also correlated with high feed efficiency in layer chickens (Yan et al., 2017). Other bacterial orders which are different between Cobb and Legacy dams include Clostridiales, Lactobacillales, and Aeromonadales, which are more abundant in the ceca of Legacy dams. Higher levels of Lactobacillales in Legacy dams may result in reduced pH levels in the cecum, as these bacteria produce lactic acid which reduces the environment’s pH (O’Hanlon et al., 2013). This environmental change may inhibit pH-sensitive *Akkermansia* bacteria (Van Herreweghen et al., 2017). This is supported by the negative correlation observed between Lactobacillales and *Akkermansia* levels. Last, we identified a relatively large variation in Lactobacillales levels in the Legacy breed (Figure 6). In an attempt to explain this variability, we also noted a large variability in Bacteroidales levels (Figure 6). Indeed, we found a negative correlation between the two (Table 1). This negative correlation was previously observed, and it was suggested that these groups have an overlapping ecological niche based on their encoded carbohydrate utilizing functions (Ma et al., 2020).

Our results show specifically that *Akkermansia* bacteria colonized Cobb but not Legacy dams. One hypothesis for this difference is that breeding programs select not only for host genetics but also for specific bacteria. If some bacteria are vertically transmitted between generations, perhaps by surviving in or on the egg and colonizing the chicks (Ding et al., 2017; Lee et al., 2019; Shterzer et al., 2020) genetic drift processes in these bacteria could result in divergent strains that are specifically adapted to the selected chicken line. Indeed, it is known that different mouse strains harbor different gut microbial communities (Jacobson et al., 2018). However, most of the bacterial composition between Cobb and Legacy dams was similar, implying this was not true for most bacterial strains. Intentional exposure of newly hatched chicks to *Akkermansia* resulted in colonization at high levels (Kubasova et al., 2019). However, *Akkermansia* were not found in young individuals sampled from other sites. It could be expected that if *Akkermansia* bacteria were common to Cobb because they were carried on or in eggs, they would flourish by marketing age. Therefore, the differences we identified between Cobb and Legacy dams are not likely dependent on the origin facility or on vertical transmission, but on genetic differences between the lines. Moreover, any differences originating from origin facility likely disappeared through cross contamination, because both lines were housed in the same shed since day of hatch. This suggests the difference in *Akkermansia* colonization between Cobb and Legacy dams stems from physiological differences affecting the gut environment.

The identification of *Akkermansia* as differentially abundant between the two lines is of interest also because this group of organisms are known to degrade mucin. Theoretically, at least two options exist to explain this difference. One is that some of the genetic changes which have occurred during the growth directed breeding program regulate mucin secretion levels and/or composition. In this case, *Akkermansia* organisms might better colonize the industrial Cobb line because they find more suitable mucin in the cecum, which they can degrade and utilize as a nutrient source. The other option is that in the less feed-efficient Legacy dams, more nutrients pass the small intestine into the cecum, allowing the creation of a bacterial community which utilizes diverse nutrient sources. In comparison, the feed-efficient Cobb dams absorb most of the feed derived nutrients in the small intestine, leaving less feed derived nutrients to reach the cecum. In such an environment, mucin degraders feeding off cecum produced mucins will be more successful. In this scenario, host mucin genetics are likely not changed; rather genetic changes that affect feed-efficiency indirectly affect cecum composition. However, other mechanisms might also affect colonization of *Akkermansia* and other bacteria. For example, differences in metabolism causing changes in body temperature may affect colonization success (Tallentire et al., 2016). Thus, further research is needed to determine whether mucin levels or composition are indeed involved in the differential levels of *Akkermansia* between slow- and fast-growing lines.

The contribution of the gut microbiota to the mechanism of action of breeding programs is unknown. Our results show that cecum microbiota composition is different between the two groups of dams. As the cecum microbial community contributes to the digestion of nutritional fibers found in the feed that the chicken cannot digest by itself (Józefiak et al., 2004), it is possible that these differences contribute to the high feed efficiency of modern commercial breeds. However, it should be noted that we have identified these differences in mature dams and not in younger poultry, which are the main target of breeding programs. Furthermore, our results show that *Akkermansia*, which specifically colonize modern breeder dams, do not colonize modern broilers at least until marketing age. Therefore, it is unclear if the identified differences in the cecum bacterial community contribute to the fast growth of modern poultry lines. We have also shown that a major difference is that *Akkermansia* organisms colonize Cobb but not Legacy dams. Indeed, *Akkermansia muciniphila* has been correlated with better feed efficiency in layers (Yan et al., 2017). On the other hand, exposure of newly hatched chicks to *A. muciniphila* seems to have a minimal impact on growth (Zhu et al., 2020).

The identification of higher levels of *Akkermansia* in Cobb dams raises an interesting question: does *Akkermansia* cause Cobb dams to divert energy from egg to mucin production? If this is the case, strategies that will limit *Akkermansia* colonization might improve egg production.

## Conclusion

In this study, we compared the composition of the gut microbiota of the current Cobb commercial breeder line and a Legacy line which has not undergone selection for 35 years. We were able to identify differences in the cecal bacterial community that were the result of genetic changes brought about by the broilers breeding programs. Specifically, Bacteria of the genus *Akkermansia* implicated in mucin degradation and associated with host metabolic health were a prominent member of the Cobb breeders’ cecum community, but were mostly absent from Legacy line dams. Inversely, Legacy dams had higher levels of Clostridiales, Lactobacillales and Aeromonadales. While we do not know if these differences also contribute to the fast growth of the current commercial line, by identifying these bacteria, we can now specifically target them for further study.

## Data Availability

The datasets presented in this study can be found in online repositories. The names of the repository/repositories and accession number(s) can be found below: https://www.ncbi.nlm.nih.gov/, PRJNA899345, PRJNA936484, PRJNA885292.
