# Supplementary material for: Differences in gut bacterial community composition between modern and slower-growing broiler breeder lines: Implications of growth selection on microbiome composition
Source: Front Physiol. 2023 Mar 21;14:1151151. doi: 10.3389/fphys.2023.1151151 (PMC10070808; doi:10.3389/fphys.2023.1151151)
Supplement: Supplementary file 1 [file Image1.pdf]

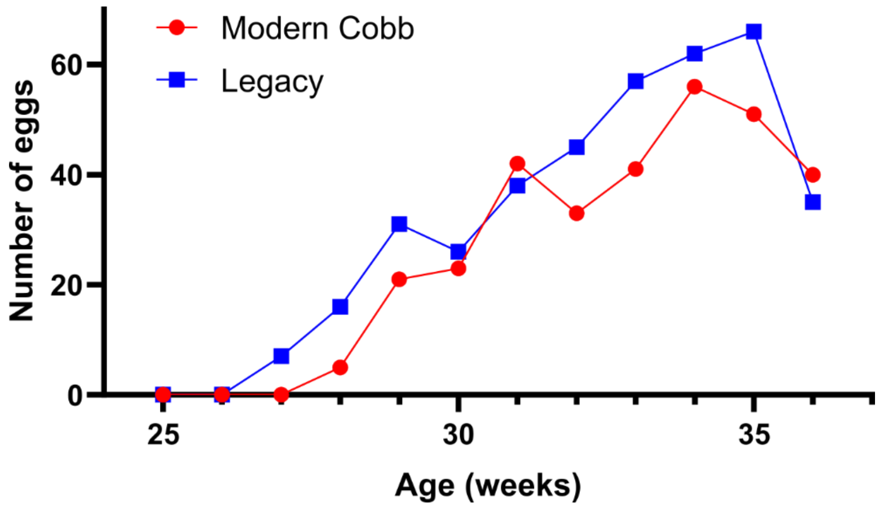


Figure S1: Laying performance of Cobb and Legacy dams. Each point represents the sum of eggs from all 10 dams over the course of one week. Dams were sampled at the beginning of week 37.
